# Naturalistic Spike Trains Drive State-Dependent Homeostatic Plasticity in Superficial Layers of Visual Cortex

**DOI:** 10.3389/fnsyn.2021.663282

**Published:** 2021-04-15

**Authors:** Varun Chokshi, Bryce D. Grier, Andrew Dykman, Crystal L. Lantz, Ernst Niebur, Elizabeth M. Quinlan, Hey-Kyoung Lee

**Affiliations:** ^1^The Zanvyl-Krieger Mind/Brain Institute, Johns Hopkins University, Baltimore, MD, United States; ^2^Cell Molecular Developmental Biology and Biophysics (CMDB) Graduate Program, Johns Hopkins University, Baltimore, MD, United States; ^3^The Solomon H. Snyder Department of Neuroscience, Johns Hopkins School of Medicine, Baltimore, MD, United States; ^4^Department of Biology, University of Maryland, College Park, MD, United States; ^5^Neuroscience and Cognitive Science Program, Brain and Behavior Institute, University of Maryland, College Park, MD, United States; ^6^The Kavli Neuroscience Discovery Institute, Johns Hopkins University, Baltimore, MD, United States

**Keywords:** metaplasticity, homeostatic plasticity, synaptic depression, visual experience, mEPSCs, miniature excitatory postsynaptic currents, Poisson stimulation

## Abstract

The history of neural activity determines the synaptic plasticity mechanisms employed in the brain. Previous studies report a rapid reduction in the strength of excitatory synapses onto layer 2/3 (L2/3) pyramidal neurons of the primary visual cortex (V1) following two days of dark exposure and subsequent re-exposure to light. The abrupt increase in visually driven activity is predicted to drive homeostatic plasticity, however, the parameters of neural activity that trigger these changes are unknown. To determine this, we first recorded spike trains *in vivo* from V1 layer 4 (L4) of dark exposed (DE) mice of both sexes that were re-exposed to light through homogeneous or patterned visual stimulation. We found that delivering the spike patterns recorded *in vivo* to L4 of V1 slices was sufficient to reduce the amplitude of miniature excitatory postsynaptic currents (mEPSCs) of V1 L2/3 neurons in DE mice, but not in slices obtained from normal reared (NR) controls. Unexpectedly, the same stimulation pattern produced an up-regulation of mEPSC amplitudes in V1 L2/3 neurons from mice that received 2 h of light re-exposure (LE). A Poisson spike train exhibiting the same average frequency as the patterns recorded *in vivo* was equally effective at depressing mEPSC amplitudes in L2/3 neurons in V1 slices prepared from DE mice. Collectively, our results suggest that the history of visual experience modifies the responses of V1 neurons to stimulation and that rapid homeostatic depression of excitatory synapses can be driven by non-patterned input activity.

## Introduction

Drastic changes in visual experience can induce long-lasting effects on excitatory synapses (Espinosa and Stryker, 2012; Cooke and Bear, 2014). In addition to inducing Hebbian plasticity, alterations in visual experience produce homeostatic plasticity of excitatory synapses in primary visual cortex (V1) of rodents (Kirkwood and Bear, 1994; Kirkwood et al., 1996; Desai et al., 2002; Goel et al., 2006; Keck et al., 2013). Specifically, in L2/3 neurons, removal of visually-driven activity by dark-exposure (DE) increases the amplitude of miniature excitatory postsynaptic currents (mEPSCs), while re-exposing DE mice to light for 2 h (LE) is sufficient to reduce mEPSC amplitudes to basal levels (Goel and Lee, 2007; Gao et al., 2010; Petrus et al., 2014; Chokshi et al., 2019). Mechanistically, the bidirectional homeostatic adaptation of excitatory synaptic strength in V1 is mediated by regulation of α-amino-3-hydroxy-5-methyl-4-isoxazolepropionic acid (AMPA) receptor function (Goel et al., 2011). Recent studies suggest that DE and LE-induced changes in excitatory synaptic transmission is input-specific to lateral intracortical inputs to L2/3 (Petrus et al., 2015; Chokshi et al., 2019), and is dependent on N-methyl-D-aspartate receptor (NMDAR) activation (Bridi et al., 2018; Chokshi et al., 2019; Rodriguez et al., 2019). These results support the idea that DE and LE-induced homeostatic plasticity of excitatory synapses are likely due to metaplasticity (Lee and Kirkwood, 2019) as proposed by the sliding threshold Bienenstock-Cooper-Monroe (BCM) model (Bienenstock et al., 1982; Bear et al., 1987; Cooper and Bear, 2012). According to the BCM model, DE would slide down the synaptic modification threshold to promote long-term potentiation (LTP), while LE would slide up the synaptic modification threshold to favor long-term depression (LTD). In this framework, DE-induced increase in mEPSCs would be a manifestation of LTP, while LE-induced reduction in mEPSCs would reflect LTD. Input-specificity and dependence on NMDAR activity of visual experience-dependent homeostatic synaptic plasticity support this interpretation (Chokshi et al., 2019; Rodriguez et al., 2019). Furthermore, consistent with the fact that sliding down of synaptic modification threshold is mediated by up-regulation of GluN2B-containing NMDARs (Quinlan et al., 1999; Philpot et al., 2001, 2003), DE-induced potentiation of mEPSCs is blocked by GluN2B specific antagonist and is dependent on spontaneous activity (Bridi et al., 2018). Although it is assumed that changes in input activity to L2/3 neurons are driving these synaptic changes, what aspect of neural activity drives homeostatic metaplasticity is currently unknown.

Hebbian plasticity is widely accepted to bring about long-term modifications to synapses as a function of the level of correlation between the activity of pre- and post-synaptic neurons (Hebb, 1949). Highly correlated neuronal activity induces LTP while low correlation drives LTD. From *ex vivo* slice experiments, the activity parameters required to induce LTP and LTD are well-characterized. Such studies have found that bursting activity or a tight temporal order of pre- then postsynaptic activity leads to synaptic strengthening, while low frequency or a post- then presynaptic order of activity leads to synaptic depression (Lisman, 1997). However, it is not known what synaptic activity profile can induce homeostatic plasticity. Based on studies of dissociated cultures where activity can be globally manipulated by pharmacological means, it was hypothesized that the average postsynaptic firing rate of a cell determines the polarity of homeostatic synaptic plasticity (Turrigiano et al., 1998; Turrigiano, 2008). However, studies in more intact circuitry have demonstrated that homeostatic plasticity can be induced in an input-specific manner (Kim and Tsien, 2008; Beique et al., 2011; Petrus et al., 2015; Chokshi et al., 2019), which suggests that postsynaptic firing rate may not be the factor that drives this form of plasticity, but rather, it may be driven by input activity. In support of this idea, maintaining the average firing rate of postsynaptic neurons by optogenetic stimulation during the blockade of synaptic activity still induces homeostatic up-regulation of excitatory synapses in cultured neurons (Fong et al., 2015). This suggests that input activity patterns may be a critical factor that triggers homeostatic changes.

In this study, we aimed to determine the temporal patterns of synaptic activity received by L2/3 neurons from feed-forward inputs that drive homeostatic plasticity. Based on the observation that a homeostatic reduction in synaptic strength is rapidly induced by re-exposing DE mice to light for 2 h, we determined that depression of synaptic strength from DE levels would be an ideal system to examine the relationship between activity patterns and homeostatic plasticity in an *ex vivo* V1 slice preparation. In order to use a more naturalistic activity pattern as occurs during LE, we first measured and analyzed single-unit spike trains recorded from V1 layer 4 (L4) of awake head-fixed DE mice re-exposed to light. When we used this *in vivo* spike train pattern to stimulate L4 in V1 slices obtained from DE mice, we observed a reduction in the amplitude of mEPSCs recorded in L2/3 neurons. Synaptic depression was also observed by a Poisson random stimulation pattern of the same average frequency. Unlike LE-induced synaptic changes, naturalistic spike pattern-driven plasticity was not dependent on GluN2B-containing NMDAR or mGluR5 activity. Interestingly, the polarity of the change was dependent on the prior visual experience of the animal.

## Materials and Methods

### Mice

Male and female mice C57BL/6 (The Jackson Laboratory) were reared in a 12 h light/12 h dark cycle. Young animals were dark exposed (DE) for 2 days (DE) between postnatal day 21 (P21) and 35 (P35). DE animals were cared for in a dark room with infrared vision goggles using dim infrared light. Some mice were re-exposed to normal light conditions for 2 h (LE) to study the effects of light exposure. All experiments were done in accordance with protocols approved by the Institutional Animal Care and Use Committees of Johns Hopkins University and University of Maryland.

### *In vivo* Single-Unit Recordings in Awake Head-Fixed Mice

Custom-made 16-channel laminar arrays were constructed and implanted as previously described (Murase et al., 2016). Briefly, a 1.2 mm 16-channel platinum-iridium electrode shank (15–20 kΩ) with a head post was implanted into the binocular region of primary visual cortex (3.00 mm lateral to the midline/0.01 mm rostral to lambda) to a depth of 1 mm, to center the electrode shank on the vertical center of cortex. For implantation, adult mice were anesthetized in 3% isoflurane in 100% O_2_. The mice received post-surgical buprenorphine (0.1 mg/kg) after return of the righting reflex and were allowed 3–4 days to recover from surgery. One day prior to recording, subjects were habituated to the head restraint for 45 min. Single unit activity was recorded in awake head restrained animals in response to square wave gratings (100% contrast, 0.05 cycles per degree, 135 orientation) presented on a CRT monitor (Clinton, 28 cm × 36 cm, 60-Hz) placed 18 cm from the eyes, subtending 75 degrees of visual space vertically and 90 degrees horizontally (+Pattern group) or a gray screen at equal luminance (27 cd/m^2^) (+Gray group).

L4 was identified as the location of the short-latency (~150 ms) visually-evoked current source in the current source density (CSD) analysis of laminar local field potential (LFP) profiles. For isolated single-unit activity, the acquired signal was filtered from 300-Hz (high pass) to 5-kHz (low pass) and sampled at 25-kHz. Unit activity was acquired for 200 trials (1 s each) using an RZ5 Bioamp Processor (TDT). Spike sorting was based on waveform shape and a Bayesian fixed variable principle component analysis using Open Sorter (TDT). Neuron classification was based on three parameters: slope of the wave for 0.5 ms after the trough, time elapsed between the trough and peak, and the ratio between the height of the trough and peak (Niell and Stryker, 2008). Only regular spiking units, defined by a low peak to trough ratio, a long duration, and a small end slope, were included for analysis.

### Spike Train Analysis

Single unit spike data were analyzed following processing with a non-biased method to delineate single spikes from bursts (Chen et al., 2009). Inter-spike intervals (ISIs) were used to generate a 1-dimensional data array from which we calculated the mean ISI value. ISIs which were greater or equal to the mean value were removed, and the remaining ISI values were used to calculate a second mean ISI for this population which was used as the threshold for detecting bursts. A burst was defined as a group of ISIs in which two or more consecutive ISIs had values that fell below the threshold. Using this method of burst detection, we wrote an algorithm in MATLAB that allowed automatic analysis of bursts and non-burst spikes from each *in vivo* recorded spike train data set. The specific algorithm used for this burst detection program and the MATLAB code is available online^1^.

The following parameters were analyzed for each spike train: average firing rate, average number of intraburst ISIs, average duration of bursts, average number of interburst ISIs, average duration of interburst intervals, mean burst frequency (inverse of mean intraburst ISI), mean non-burst frequency (inverse of mean interburst ISI), and fraction of spikes in bursts [(Number of ISIs in burst)/(Total number of ISIs per spike train)].

### Generation of Synthetic Spike Train With Poisson Distribution

A synthetic spike train was generated in MATLAB using a Poisson process with the mean ISI set equal to the average ISI of the +Gray group (see Tables s 1, 2).

**Table 1:** Spike train properties analyzed from *in vivo* primary visual cortex (V1) layer 4 (L4) cells.

		LE groups	
Spike train property	DE	+Gray stimulation	+Pattern stimulation
Average firing rate (Hz)	2.74 ± 0.32	5.52 ± 0.73**	5.82 ± 0.71**
Mean burst length (# of spikes/burst)	5.82 ± 0.34	11.61 ± 1.82**	10.12 ± 1.60*
Mean burst frequency (Hz)	33.17 ± 1.86	54.28 ± 10.56	51.30 ± 11.75
Mean non-burst frequency (Hz)	1.39 ± 0.16	2.34 ± 0.49*	2.56 ± 0.36*
Frequency of bursts (Hz)	0.30 ± 0.04	0.34 ± 0.05	0.40 ± 0.04
Fraction of spikes in bursts	0.61 ± 0.01	0.68 ± 0.04	0.66 ± 0.04

Statistics: ANOVA with Newman–Keuls multiple comparison test; **p < 0.01, *p < 0.05.

**Table 2:** Spike train properties of the exemplar cells used for *ex vivo* stimulations.

Spike train property	+Gray stimulation	+Pattern stimulation	Poisson stimulation
Average firing rate (Hz)	4.77	4.05	5.52
Mean burst length (# of spikes/burst)	9.45	7.91	2.38
Mean burst frequency (Hz)	46.40	44.12	43.04
Mean non-burst frequency (Hz)	1.64	1.60	5.35
Frequency of bursts (Hz)	0.39	0.37	0.14
Fraction of spikes in bursts	0.76	0.71	0.06

### Acute Slice Preparation for mEPSC Recording

The mice were anesthetized by isoflurane vapors and decapitated after verifying the absence of a toe-pinch response. Brain blocks containing visual cortex were coronally sliced into 300-μm sections using a vibratome (Leica VT1200S or Ted Pella Pelco easislicer^TM^) in ice-cold dissection buffer containing 212.7 mM sucrose, 10 mM dextrose, 3 mM MgCl_2_, 1 mM CaCl_2_, 2.6 mM KCl, 1.23 mM NaH_2_PO_4_•H_2_O, and 26 mM NaHCO_3_, which was bubbled with a 95%-O_2_/5%-CO_2_ gas mixture. The slices were incubated at room temperature for 60 min in artificial cerebrospinal fluid (ACSF: solution containing 124 mM NaCl, 5 mM KCl, 1.25 mM NaH_2_PO_4_•H_2_O, 26 mM NaHCO_3_, 10 mM dextrose, 2.5 mM CaCl_2_, and 1.5 mM MgCl_2_, bubbled with 95% O_2_/5% CO_2_).

### Whole-Cell Voltage-Clamp Recordings of mEPSCs

Visual cortical slices were transferred to a submersion-type recording chamber mounted on the fixed stage of an upright microscope with oblique illumination and were continually supplied with ACSF bubbled with 5% CO_2_/95% O_2_(30°C) with a flow rate of approximately 2 ml/min. AMPA receptor-mediated mEPSCs were isolated by adding 1 μM tetrodotoxin (TTX), 20 μM bicuculline (Bic), and 100 μM DL-2-amino-5-phosphonopentanoic acid (APV). Recording pipettes were filled with internal solution containing: 130 mM Cs-gluconate, 10 mM HEPES, 8 mM KCl, 1 mM EGTA, 4 mM disodium-ATP (Sigma–Aldrich, Cat #A6419), 10 mM disodium-phosphocreatine (Sigma–Aldrich, Cat #P7936), 0.5 mM Sodium-GTP (Sigma–Aldrich, Cat #G8877), 5 mM Lidocaine N-ethyl bromide (Sigma–Aldrich, Cat #L5783). Biocytin (1 mg/ml) was added to the internal solution to confirm morphology and location of the recorded cells *post hoc*. Pyramidal neurons in L2/3 of V1 were recorded in voltage-clamp at −80 mV and the recorded mEPSCs were digitized at 10-kHz by a National Instruments data acquisition board and acquired through an Igor based program (Wavemetrics). Two-hundred mEPSCs were analyzed from each cell with Mini Analysis (Synaptosoft).

### *Ex vivo* Stimulations to Induce Plasticity

Visual cortical slices were transferred to a submersion-type recording chamber mounted on the fixed stage of an upright microscope with oblique infra-red illumination. Slices were continually supplied with ACSF bubbled with 5% CO_2_/95% O_2_ (30°C) with a flow rate of about 2 ml/min. Pyramidal neurons in L2/3 of V1 were recorded in the whole-cell configuration. To generate activity patterns, a bipolar stimulation electrode was placed in V1 L4. Prior to stimulation a V1 L2/3 pyramidal cell was recorded under voltage clamp at −80 mV to determine the intensity of stimulation from L4. The stimulus intensity was adjusted using a stimulation isolated unit (SIU91A, Cygnus Instruments) to produce EPSCs of approximately 200 pA in the V1 L2/3 cell. The recording was then switched to the current clamp configuration, and we confirmed that the L4 stimulus results in subthreshold EPSPs. A small holding current was injected to keep the membrane potential at −65 mV in current-clamp, and Igor NIDAQ Tools MX was used to generate the desired stimulation pattern for 2 h.

### Statistics

All data is displayed as Mean ± S.E.M. Normality was confirmed with the D’Agostino and Pearson normality test. Unpaired *t*-tests and ANOVAs were performed to compare the averages of two or more normally distributed data sets, respectively. Data sets that were not normally distributed were compared using non-parametric statistical tests (e.g., Mann-Whitney or Kruskal–Wallis). Cumulative distribution of individual mEPSC amplitudes were compared using the Kolmogorov–Smirnov test. Statistical analyses were performed in Prism (Graphpad). *P* < 0.05 was taken as a statistically significant difference on all tests, except for Kolmogorov–Smirnov tests in which we used *P* < 0.001 as the cutoff.

## Results

### Analysis of V1 L4 Neuronal Spiking Activity Recorded During LE

**Figure 1:**
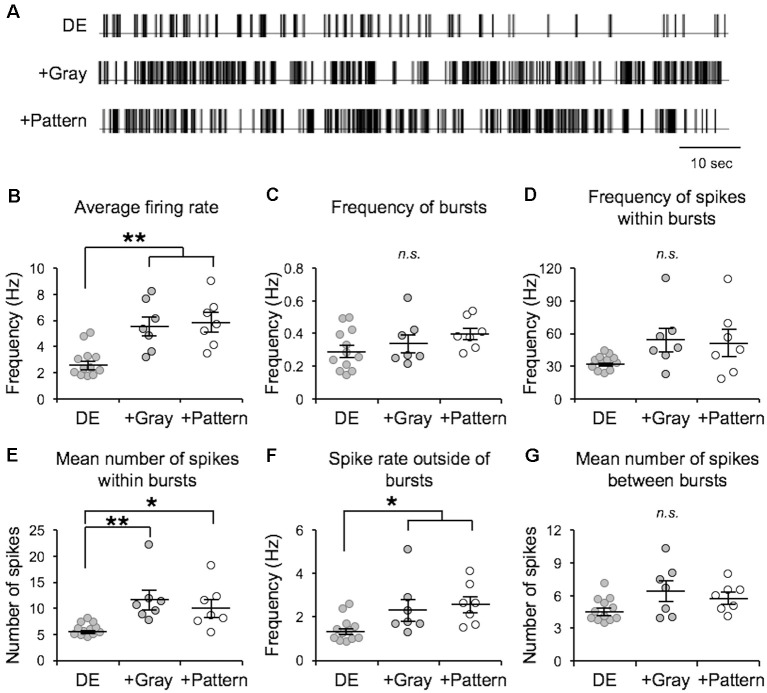
Analysis of visual experience-dependent *in vivo* spike activity. Single unit recordings were acquired in primary visual cortex (V1) layer 4 (L4) of awake head-fixed dark exposed (DE) mice facing a blank screen in a dark room (DE), DE mice placed in a lighted room facing a gray screen (+Gray), and DE mice placed in a lighted room facing a screen with a grating (+Pattern). See “Materials and Methods” section for details. **(A)** Example inter-spike-interval trains recorded from DE (top), +Gray (middle), and +Pattern (bottom) conditions. Each vertical line represents an action potential. **(B)** Average overall firing rate was significantly elevated in the two groups receiving visual stimuli (DE = 2.74 ± 0.32 Hz; DE+Gray = 5.52 ± 0.73 Hz; DE+Pattern = 5.82 ± 0.70 Hz; ANOVA, *F* = 1.341, *p* = 0.0004; Newman–Keuls multiple comparison **p < 0.01). **(C)** There was no significant difference in the frequency of bursts (see “Materials and Methods” section for quantification of bursts) across groups (DE = 0.295 ± 0.036 Hz; DE+Gray = 0.341 ± 0.054 Hz; DE+Pattern = 0.397 ± 0.037 Hz; ANOVA, *F* = 1.511, *p* = 0.2417). n.s.: not statistically significant. **(D)** There was an increase in the average frequency of spikes within a burst in the two groups receiving visual stimuli, which did not reach statistical significance (DE = 33.17 ± 1.86 Hz; DE+Gray = 54.28 ± 10.56 Hz; DE+Pattern = 51.30 ± 11.75 Hz; ANOVA, *F* = 2.647, *p* = 0.0923). This was due to a large increase in variance (Bartlett’s test for standard deviations, Bartlett’s *statistic* = 18.4, *p* = 0.0001). **(E)** The mean number of spikes within a burst was significantly increased in the visual stimuli groups (DE = 5.82 ± 0.34; DE+Gray = 11.61 ± 1.82; DE+Pattern = 10.12 ± 1.60; ANOVA, *F* = 7.575, *p* = 0.0030; Newman–Keuls multiple comparison *p < 0.05, **p < 0.01). **(F)** There was a significant increase in the frequency of spikes outside of bursts in the two visual stimuli groups (DE = 1.39 ± 0.16; DE+Gray = 2.35 ± 0.49; DE+Pattern = 2.56 ± 0.36; ANOVA, *F* = 4.472, *p* = 0.0229; Newman–Keuls multiple comparison *p < 0.05). **(G)** There was no significant difference in the mean number of spikes between bursts across the three groups (DE = 4.63 ± 0.31; DE+Gray = 6.40 ± 0.90; DE+Pattern = 5.78 ± 0.49 ANOVA, *F* = 3.043, *p* = 0.0672).

In the canonical feedforward pathway, visually driven activity is conveyed to L2/3 neurons by excitation from thalamo-recipient L4 neurons. Hence, we aimed to test whether activating L4 with naturalistic neural activity associated with LE would drive metaplasticity in V1 L2/3 principal neurons. To identify activity patterns (neuronal spiking activity) in V1 L4 neurons with LE, we recorded single-unit activity in awake mice during two hours of re-exposure to light following two days of DE. Single unit recordings from V1 L4 regular spiking neurons were acquired in awake head-fixed mice *via* a chronically implanted multielectrode array. Recordings were acquired from DE mice during presentation of a blank screen (no visual stimulation) and during LE in response to a homogenous gray screen (+Gray) or a patterned visual stimulus (equiluminant high contrast grating; +Pattern). The *in vivo* spike trains recorded from the three groups (DE, +Gray, and +Pattern) were analyzed to extract various metrics of spike patterns. We used an ISI-based spike train analysis method (Chen et al., 2009) to distinguish spike bursts from non-burst spike activity (Table 1 and Figure 1). There was no statistically significant difference in the measured firing properties of V1 L4 neurons from when mice viewed the visual stimulus (+Pattern) or the gray screen (+Gray; Figure 1). The average firing rate (overall firing rate), mean burst length (mean number of spikes within bursts), and mean non-burst frequency (spike rate outside of bursts) were significantly higher in the +Gray and +Pattern LE groups (Figure 1 and Table 1). The spike rate within identified burst periods trended towards an increase in the LE groups, but did not reach a statistical significance likely due to the increase in variance (Figure 1D and Table 1). There was no statistically significant increase in the overall frequency of bursts across the three groups (Figure 1C and Table 1). Furthermore, there were no statistical differences in the metrics of spike patterns of V1 L4 neurons between +Gray and +Pattern groups (Figure 1 and Table 1). Our results demonstrate that the increase in visual evoked activity during LE is largely due to an increase in burst length and non-burst spike rates.

### *In vivo* LE Spike Patterns Reduce mEPSC Amplitude in V1 L2/3 of DE Mice *Ex vivo*

**Figure 2:**
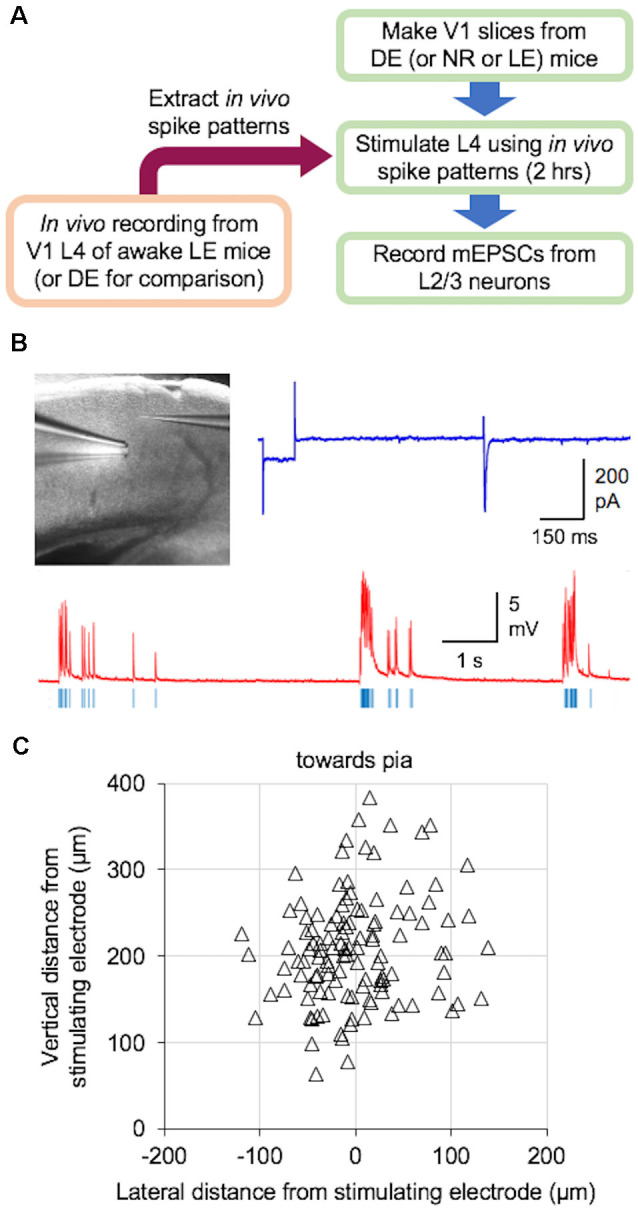
*Ex vivo* paradigm to examine plasticity outcome following naturalistic spike pattern stimulation. **(A)** Schematic diagram of the experiments. *In vivo* spike trains were recorded from V1 L4 of awake head-fixed mice that were dark-exposed and brought out to light (LE) to view drifting gratings (+Pattern) or an equal luminance gray screen (+Gray). A group of DE mice was recorded in the dark (DE) for comparison. Recorded spike trains were analyzed to extract inter-spike-interval (ISI) information, which was then used to construct the stimulus train. V1 slices were obtained from DE [or normal reared (NR) or LE] mice. L4 of these slices were stimulated with the *in vivo* spike pattern for 2 h. Stimulus intensity was adjusted to produce subthreshold activation of L2/3 neurons (see **B**). After the 2-h stimulation, miniature excitatory postsynaptic currents (mEPSCs) were recorded from L2/3 pyramidal cells under whole-cell voltage clamp. **(B)** *Ex vivo* experimental setup in acute V1 slice preparation. Top left: image of a V1 slice with a stimulating electrode in L4 and a whole-cell recording electrode patched onto a L2/3 pyramidal cell. Top right: an example voltage-clamp trace recorded when determining the stimulation intensity, which was adjusted to induce approximately 200 pA evoked excitatory postsynaptic currents (EPSCs) in V1 L2/3 neurons. Bottom: an example current-clamp recording trace while delivering an *in vivo* pattern of activity (red, current-clamp trace from V1 L2/3 neuron; blue vertical lines, stimulation delivered to an electrode in L4). Stimulation was done for 2 h. **(C)** Distribution of recorded V1 L2/3 neuron location (open triangles) relative to the location of the stimulating electrode in L4 (0,0 coordinate).

**Figure 3:**
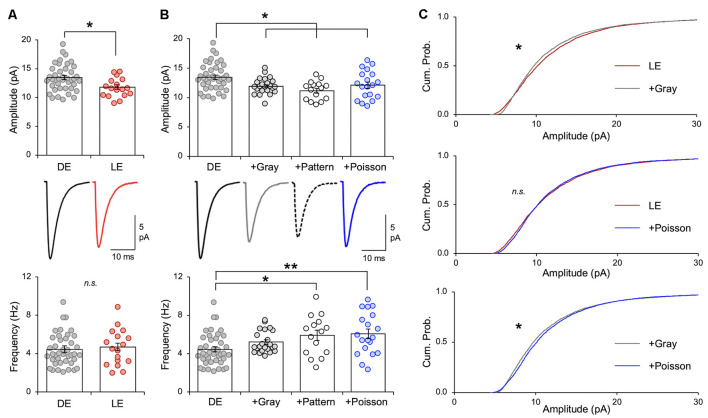
Naturalistic spike patterns induce homeostatic synaptic depression. **(A)** LE decreases excitatory synaptic strength in mouse V1 L2/3 pyramidal neurons, as reported previously (Goel and Lee, 2007; Gao et al., 2010). Comparison of average mEPSC amplitude from V1 L2/3 pyramidal neurons following 2 days of DE and a subsequent 2 h of LE in P23–35 mice. Top: average mEPSC amplitude from individual cells are shown as circles and mean ± S.E.M. are shown as bars (DE = 13.46 ± 0.36 pA, *n* = 41, LE = 11.81 ± 0.40 pA, *n* = 17; unpaired *t*-test, *t* = 2.716, **p = 0.0088). Middle: average mEPSC traces. Bottom: there was no change in average mEPSC frequency (DE median = 4.021 Hz, LE median = 4.592 Hz; Mann–Whitney test, *U* = 330, *p* = 0.761). **(B)** Top: comparison of average mEPSC amplitudes from different stimulation regimes. mEPSCs were recorded from the following groups: V1 slices from DE mice (DE), V1 slices from DE mice stimulated with *in vivo* spike patterns from exemplar cells of LE (+Gray; +Pattern), and V1 slices from DE mice stimulated with a Poisson random train (+Poisson). Average mEPSC amplitudes of individual cells are plotted as circles and mean ± S.E.M are shown as bars (DE: the same data shown in panel **A** are replotted here for comparison; DE+Gray = 11.93 ± 0.28 pA, *n* = 23; DE+Pattern = 11.18 ± 0.41 pA, *n* = 15; DE+Poisson = 12.11 ± 0.55 pA, *n* = 19; ANOVA, *F* = 5.138, *p* = 0.0025; Newman–Keuls multiple comparison test, *p < 0.05). Middle: average mEPSC traces. Bottom: comparison of average mEPSC frequency across groups (DE median = 4.021 Hz; DE+Gray = 4.753 Hz; DE+Pattern = 6.298 Hz; DE+Poisson = 5.695 Hz; Kruskal—Wallis statistic = 13.47, *p* = 0.0037; Dunn’s multiple comparison test, *p < 0.05, **p < 0.01). **(C)** Top: cumulative probability of mEPSC amplitudes of LE (red line) and +Gray (gray line) group. There is a significant difference between the two groups (Kolmogorov–Smirnov test: *p < 0.0001). Middle: cumulative probability of mEPSC amplitudes of LE (red line) and +Poisson (blue line) group. There was no statistical difference in the distribution (n. s.). Bottom: cumulative probability of mEPSC amplitudes of +Gray (gray line) and +Poisson (blue line) group. There is a significant difference between the two groups (Kolmogorov–Smirnov test: *p < 0.0001).

We examined if naturalistic spike patterns obtained from V1 L4 neurons during LE could drive homeostatic reduction in the strength of excitatory synapses onto L2/3 neurons. To do this, we stimulated L4 of V1 slices obtained from DE mice with the *in vivo* spike patterns recorded from L4 of LE mice (+Gray and +Pattern conditions; Figure 2). For both +Gray and +Pattern, the cell with the average firing rate closest to the group mean was chosen as the exemplar to provide the stimulation pattern for that group (Table 2). For each experiment, we first determined the stimulation intensity that was sufficient to evoke an approximately 200 pA EPSC in a L2/3 neuron (Figure 2B). We determined the stimulation intensity based on reports that visual stimulus presentation results in EPSCs of roughly 100–500 pA in V1 L2/3 pyramidal neurons recorded *in vivo* at similar holding potentials in the whole-cell voltage-clamp configuration (Sun and Dan, 2009; Haider et al., 2016). We found that the stimulation intensity, generated subthreshold EPSPs in current-clamp (Figure 2B). L4 was then stimulated at the determined intensity with the +Gray or +Pattern exemplar cell activity for 2 h to match the duration of LE shown to produce a reduction in mEPSC amplitudes in L2/3 neurons without changes in mEPSC frequency (Figure 3A). Following stimulation with either +Gray or +Pattern activity, mEPSCs were recorded from L2/3 neurons directly above the L4 stimulating electrode (Figure 2C).

Stimulation of DE slices with the spike pattern of the +Gray exemplar cell (see Table 2 for parameters) significantly decreased the average mEPSC amplitude in L2/3 neurons (Figure 3B). We saw no significant change in mEPSC frequency in the *ex vivo* stimulated slices. While the decrease in mEPSC amplitude on average looks similar to what we observed with LE (Figure 3A), the cumulative probability of mEPSC amplitudes from LE and +Gray conditions were statistically different from each other (Figure 3C). This suggests that plasticity induced by LE and +Gray is either not the same across synapses or that they are affecting a non-identical subset of synapses. Stimulating DE slices with the spike pattern of the +Pattern exemplar cell (see Table 2 for parameters) also produced similar results as the +Gray group, except this was accompanied by a significant increase in mEPSC frequency. To control for possible confounding effects of prolonged exposure to the perfusion bath prior to recording mEPSCs, a subset of DE slices was placed in the recording chamber for 2 h with a stimulating electrode, but received no stimulation (+No Stim) before recording. We observed no difference in average mEPSC amplitude or frequency between the +No Stim and DE control (amplitude: DE = 13.08 ± 0.35 pA, *n* = 41; DE+No stim = 13.08 ± 0.54 pA, *n* = 22; Unpaired *t-test*, *t* = 0.0634, *p* = 0.5485; frequency: DE median = 4.021 Hz; DE+No stim median = 4.368 Hz; Mann–Whitney test, *U* = 391, *p* = 0.3939). These data demonstrate that stimulating V1 L4 of DE mice with naturalistic spiking patterns obtained from L4 neurons during LE is sufficient to reduce the amplitude of mEPSCs in L2/3 neurons. Given that our stimulation is subthreshold for postsynaptic spike generation, these data suggest that the average frequency of input activity onto a given cell, not the postsynaptic firing rate, is the key factor driving homeostatic weakening of excitatory synapses in V1 L2/3 neurons.

### Stimulation With a Poisson Spike Train Mimics the Effect of Naturalistic LE Spike Patterns

**Figure 4:**
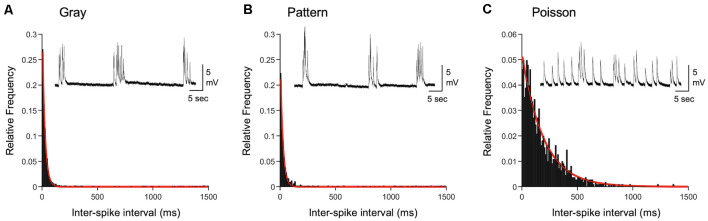
Comparison of inter-spike intervals of naturalistic spike trains. **(A)** Histogram of inter-spike intervals (ISIs) recorded from L4 of a DE mouse exposed to light and viewing a gray screen (corresponding to +Gray exemplar cell in Table 2). The histogram is fitted with a single exponential decay function (red line). Inset: an example current-clamp recording trace from a V1 L2/3 neuron of a DE mouse. Stimulation train from the +Gray exemplar cell was delivered through an electrode placed in L4 of the slice. **(B)** Histogram of ISIs recorded from L4 of a DE mouse exposed to light and viewing a screen with patterned gratings (corresponding to +Pattern exemplar cell in Table 2). The histogram is fitted with a single exponential decay function (red line). Inset: an example current-clamp recording from a V1 L2/3 neuron of a DE mouse receiving a train of +Pattern stimulation through an electrode in L4. **(C)** Histogram of ISIs of a Poisson train with the same average firing frequency as the exemplar neuron of the Gray group. The histogram is fitted with a single exponential decay function (red line). Inset: an example current-clamp recording from a V1 L2/3 neuron of a DE mouse while delivering a train of +Poisson stimulation (see Table 2 for details) to L4. Note that the stimulation intensity used generates subthreshold EPSPs. Distribution of ISIs between Gray and Pattern is not statistically significant (Kolmogorov–Smirnov test: *D* = 0.0776, *p* = 0.009), while that of Poisson is significantly different from those of Gray (Kolmogorov–Smirnov test: *D* = 0.5530, *p* < 0.0001) and Pattern (Kolmogorov–Smirnov test: *D* = 0.4893, *p* < 0.0001).

The average firing rate *in vivo* of V1 L4 neurons was higher in DE +Gray and +Pattern groups compared to DE alone (Figure 1B and Table 1), thus we hypothesized that the average firing rate of presynaptic neurons was a key factor in engaging homeostatic downregulation of synaptic strength. To test this, we wanted to manipulate the average firing rate in the absence of other aspects of patterned neuronal activity, such as bursts. We therefore generated a Poisson distribution of ISI values with a mean equivalent to the average ISI of the +Gray group, and sampled a series of ISIs from this distribution to use as a stimulation pattern (Figure 3C and Tables s 1, 2). Because the average firing rate of +Gray and +Pattern group are similar (Figure 1B and Table 2), our Poisson spike train would reflect the average firing rate of both LE conditions. We confirmed that the distribution of ISI of the Poisson spike train is different from those of +Gray and +Pattern (Figure 4). Stimulating V1 L4 of DE slices with the Poisson spike train induced a significant reduction in mEPSC amplitude in L2/3 neurons similar to what we observed with naturalistic pattern stimulations (Figure 3B). However, the cumulative probability distribution of mEPSC amplitudes in the +Poisson group was significantly different from that seen in the naturalistic pattern stimulated groups (Figure 3C). This suggests that Poisson random activity is sufficient to produce homeostatic depression of mEPSC amplitudes, even though the changes across individual synapses are not identical to those produced by naturalistic patterned stimuli. There was also an increase in mEPSC frequency following Poisson stimulation, which mirrors what we observed with the +Pattern exemplar cell (Figure 3B).

### Effect of GluN2B and mGluR5 Antagonists on the Expression of *In vivo* Spike Train-Induced Plasticity of mEPSCs

**Figure 5:**
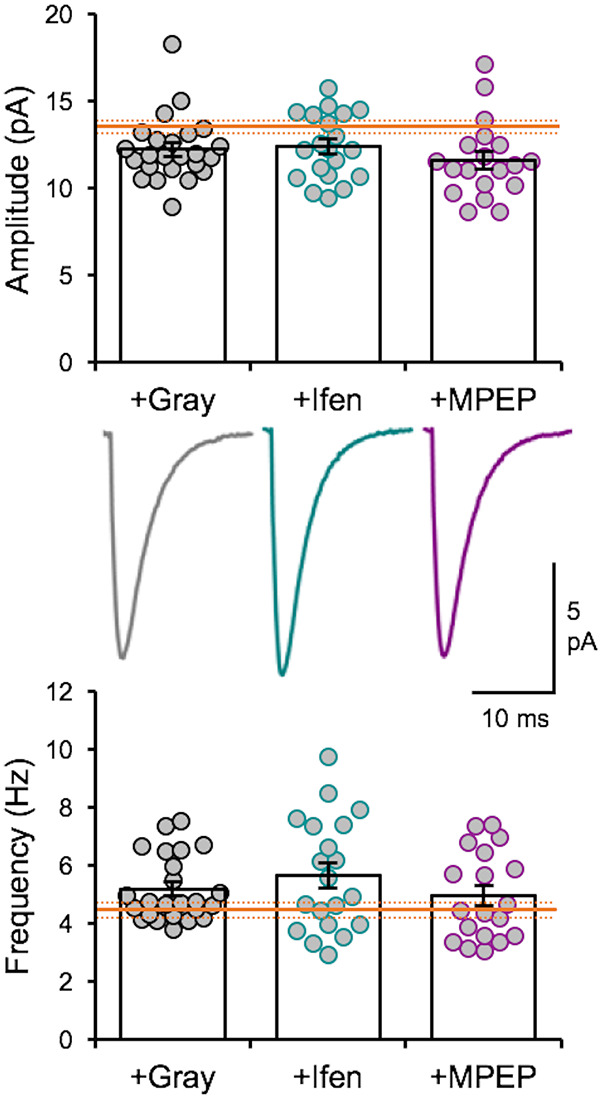
Naturalistic pattern stimulation-induced synaptic depression is not dependent on GluN2B-containing NMDARs or mGluR5s. Stimulation with +Gray activity while blocking GluN2B-containing NMDARs with ifenprodil (+Ifen) or mGluR5s with MPEP (+MPEP) did not yield significantly different changes from stimulation with +Gray alone. Top: average mEPSC amplitudes of individual neurons are shown as circles and mean ± S.E.M are shown as bars (DE+Gray = 11.93 ± 0.28 pA; DE+Gray+Ifen = 12.36 ± 0.42 pA; DE+Gray+MPEP = 11.6 ± 0.51 pA; ANOVA, *F* = 0.8522, *p* = 0.4317). For reference, the mean mEPSC amplitude for DE is shown as the orange line with ± S.E.M. shown in dotted orange lines. Middle: average mEPSC traces for each group. Bottom: average mEPSC frequency comparison across groups (DE+Gray median = 4.753 Hz; DE+Gray+Ifen median = 5.258 Hz; DE+Gray+MPEP median = 4.454Hz; ANOVA, *F* = 1.076, *p* = 0.3475). For reference, the mean mEPSC frequency for DE is shown as the orange line with ± S.E.M. shown in dotted orange lines.

We recently reported that the LE-induced reduction of mEPSC amplitudes is dependent on NMDAR and metabotropic glutamate receptor 5 (mGluR5) activity (Chokshi et al., 2019; Rodriguez et al., 2019). Therefore, we examined whether the naturalistic spike train-induced depression of mEPSCs presented here is also dependent on similar signaling. We stimulated slices from DE mice with the +Gray exemplar cell activity pattern (Table 2) in the presence of either ifenprodil (3 μM) or 2-methyl-6-(phenylethynyl)pyridine (MPEP, 50 μM) to block GluN2B-containing NMDARs or mGluR5s, respectively. Neither of the pharmacological antagonist prevented the reduction in average mEPSC amplitude driven by +Gray stimulation delivered to L4 (Figure 5). Our results suggest that the molecular signaling required for synaptic depression induced with naturalistic spike trains is different from those required for synaptic depression with LE. This finding together with our observation that +Gray results in a different shift in mEPSC amplitude distribution when compared to that of LE (Figure 3C) suggests that the plasticity induced by naturalistic spike pattern stimulation is not identical to that seen with LE.

### The Outcome of Naturalistic Spike Pattern Stimulation Is Dependent on the History of Visual Experience

**Figure 6:**
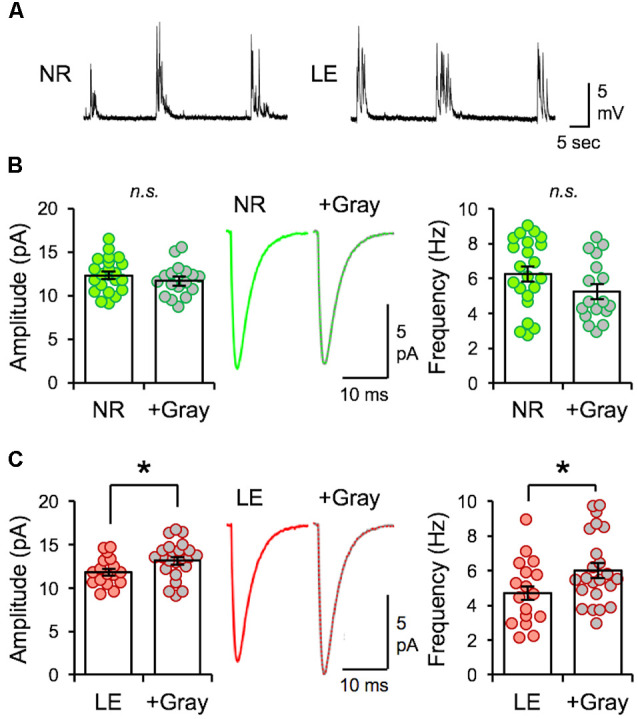
The same naturalistic pattern stimulation yields distinct synaptic changes dependent on prior visual experience. **(A)** Example current-clamp recording traces recorded from V1 L2/3 neurons while stimulating L4 with +Gray spike train in slices obtained from NR (left) and LE (right) mice. Stimulation intensity used produced subthreshold EPSPs. **(B)** No difference in the average amplitude of mEPSCs recorded from NR slices stimulated with the +Gray activity when compared to unstimulated NR slices. Left: average mEPSC amplitudes of individual neurons are shown as circles and mean ± S.E.M are shown as bars (NR = 12.32 ± 0.42 pA, *n* = 22; NR+Gray = 11.7 ± 0.44 pA, *n* = 17; unpaired *t*-test: *t* = 1.003, *p* = 0.3222). n.s.: not statistically significant. Middle: average mEPSC traces. Right: average mEPSC frequency comparison (NR = 6.26 ± 0.43 Hz, NR+Gray stim = 5.24 ± 0.43 Hz, unpaired *t*-test, *t* = 1.643, *p* = 0.1088). n.s.: not statistically significant. **(C)** A significant increase in average amplitude of mEPSC recorded from LE slices stimulated with the +Gray activity when compared to unstimulated LE slices (LE). Left: average mEPSC amplitudes of individual neurons are shown as circles and mean ± S.E.M are shown as bars (LE = 11.81 ± 0.40 pA, *n* = 17; LE+Gray = 13.15 ± 0.44 pA, *n* = 23; unpaired *t*-test: *t* = 2.716, *p* = 0.0088). Middle: average mEPSC traces. Right: average mEPSC frequency comparison (LE = 4.70 ± 0.45 Hz, LE+Gray = 6.02 ± 0.43 Hz; unpaired *t*-test, *t* = 2.084 *p = 0.0440).

Next, we examined whether the naturalistic spike pattern stimulation-induced reduction of mEPSC amplitudes in L2/3 neurons is dependent on the initial state of V1 circuitry, as determined by prior visual experience. To test this, we stimulated V1 L4 of slices obtained from normal reared (NR) control animals (NR slices) with spike pattern of the +Gray exemplar cell (Figure 6A). In contrast to the decrease in mEPSC amplitudes observed with DE slices, 2 h of +Gray stimulation in NR slices did not induce significant changes in the average amplitude or frequency of mEPSCs in L2/3 neurons (Figure 6B). However, the same spike pattern (+Gray) delivered to V1 L4 of slices from LE mice (2 h of light re-exposure after 2 days of DE; Figure 6A) induced a significant increase in both the average mEPSC amplitude and frequency (Figure 6C). Thus, the history of prior visual experience impacts the response of V1 circuit following stimulation of feedforward inputs from L4 with the same naturalistic spike pattern.

## Discussion

We found that visual experience during LE elevated the overall firing rate of V1 L4 neurons, which was primarily driven by an increase in the non-burst firing rate and an increase in mean burst length (Figure 1). By using naturalistic spike trains recorded from V1 L4 of a LE mouse to stimulate L4 of *ex vivo* V1 slices obtained from DE mice, we were able to induce homeostatic depression of mEPSC amplitudes in L2/3 pyramidal neurons (Figure 3). Homeostatic synaptic depression was also obtained with a Poisson random spike train with an average firing rate matched to the naturalistic spike train (Figure 3). While *ex vivo* stimulation with naturalistic spike trains was effective at driving depression of excitatory synaptic transmission similar to that observed with LE, it did not share signaling pathways with LE-induced plasticity (Figure 5). Interestingly, homeostatic response to *ex vivo* stimulation was dependent on the nature of prior visual experience, as demonstrated by the observation that using the same naturalistic spike train to stimulate V1 slices obtained from NR and LE mice yielded no change or an increase in mEPSC amplitudes, respectively (Figure 6).

## Average Input Activity as a Determinant for Homeostatic Metaplasticity

Bursting activity is believed to provide more robust and reliable synaptic transmission than single spikes (Lisman, 1997). It is especially important for inducing Hebbian plasticity where brief bursts of high frequency stimulation (50–100 Hz) induce robust LTP (Bliss and Gardner-Medwin, 1973; Bliss and Lomo, 1973). Conversely, prolonged stimulation at lower frequencies (1–3 Hz) induces LTD at excitatory synapses (Dudek and Bear, 1992). Here, we sought to elucidate which properties of neuronal activity drive the homeostatic metaplasticity observed in V1 following increases in visual experience. We found that V1 L4 neurons recorded *in vivo* from LE mice showed an elevated average firing rate compared to that of DE mice. This difference was largely driven by an increase in the non-burst firing rate and the average length of bursts (Figure 1). *Ex vivo* stimulation of V1 L4 in slices obtained from DE mice using naturalistic spike patterns obtained from V1 L4 neurons in LE mice was sufficient to reduce mEPSC amplitudes in L2/3 neurons, but the details of the change are not identical to what is observed in LE mice. We used stimulation intensities that produced subthreshold postsynaptic responses (Figures 2, 3), hence our results suggest that homeostatic synaptic depression can be induced by monitoring synaptic input activity rather than postsynaptic spiking activity.

While there were some differences in the plasticity of mEPSCs resulting from naturalistic pattern stimulation *ex vivo* and LE (Figures 3, 5), our results suggest that feedforward activity from L4 is able to drive synaptic depression in L2/3 as predicted with homeostatic plasticity. The differences in mEPSC regulation between *ex vivo* stimulation and LE may stem from many factors. One possibility is that the electrical stimulation given here may produce synchronized activity in the slices, which is not likely to occur with visual experience *in vivo*. Another possibility is that stimulation of L4 may not activate all of the inputs onto L2/3 neurons that visual experience does. A third possibility is that inputs other than L4 may participate in homeostatic plasticity driven by LE. There is evidence that changes in mEPSCs reflect mainly the plasticity of lateral intracortical synapses onto L2/3 neurons in V1 (Petrus et al., 2015; Chokshi et al., 2019). It is likely that these intracortical synapses will be active *in vivo* with LE, but we are unable to mimic this with our feedforward stimulation paradigm. A fourth possibility is that neurons in *ex vivo* slices tend to be relatively hyperpolarized with minimum spontaneous firing, hence may not fully recapitulate the situation *in vivo* where neurons are often in an “up-state” closer to action potential thresholds.

We found that simply presenting a Poisson random stimulation train with the same average frequency as that of V1 L4 neurons recorded from LE mice was sufficient to drive homeostatic weakening of mEPSCs in L2/3 neurons (Figure 3). This suggests that L2/3 neurons are likely computing the average input frequency, rather than specific patterns of activity, to induce homeostatic plasticity. It is of interest to note that previous studies have used Poisson random stimulation in V1 L4, but with different outcomes than what we report here (Perrett et al., 2001; Guo et al., 2012). This likely reflects the fact that the plasticity is dependent on the metaplastic state of synapses. Here, we have clearly shown that the same *in vivo* spike train results in either depression, no change, or potentiation of mEPSC amplitudes depending on the prior visual experience of the mouse (Figures 3, 6). One study that failed to induce plasticity with a Poisson random stimulation train was performed in V1 of NR guinea pig (Perrett et al., 2001). This is similar to our finding that delivering naturalistic pattern of stimulation to V1 slices from NR mice does not produce changes in mEPSCs (Figure 5B), which suggests that prior visual experience is an important variable to consider when assessing plasticity. Poisson random stimulation has previously been shown to be effective at inducing LTD in V1 of DE rats (Guo et al., 2012), but in contrast to what we have shown here, it was completely blocked by the GluN2B antagonist, ifenprodil. The difference may be due to the use of different frequency and duration of Poisson train: Guo et al. used a shorter duration (10 min) 0.5-Hz Poisson train, which is a lower frequency than what we used here (Table 2). It is possible that a short duration lower frequency Poisson train may act on GluN2B-containing NMDARs, which are up-regulated by DE (Quinlan et al., 1999; Philpot et al., 2001), to produce LTD. Our results would add to this finding by demonstrating that a more prolonged (2 h) Poisson train stimulation, which has the same average frequency as neural activity seen during LE, induces GluN2B-independent homeostatic plasticity.

## The Polarity of Plasticity Induced by the Same Naturalistic Stimulation Pattern Is Dependent on the Prior Visual Experience

The threshold for inducing Hebbian plasticity is adjusted by changes in visual experience (Abraham, 2008; Cooper and Bear, 2012). An altered synaptic modification threshold can lead to opposite polarities of synaptic changes even with activity of the same input frequency. Our observation that the same naturalistic spike train produces distinct changes to mEPSC amplitudes depending on prior visual experience (Figure 6) is consistent with the idea that prior visual experience changes the metaplastic state of V1 circuitry. Further, it suggests that naturalistic spike patterns that induce homeostatic adaptation act on the existing metaplastic state to drive synaptic adaptation.

Metaplasticity is largely driven by changes in the function of NMDA receptors, which result from alterations in subunit composition (GluN2B and GluN2A ratio). Bidirectional regulation of NMDAR subunit composition with DE and LE is well established (Quinlan et al., 1999; Philpot et al., 2001, 2003; Guo et al., 2012). DE reduces the threshold for LTP such that previously weak inputs are able to be potentiated (Kirkwood et al., 1996; Guo et al., 2012). We recently reported that in V1 L2/3 the reduction of mEPSC amplitudes with LE is dependent on NMDARs (Chokshi et al., 2019; Rodriguez et al., 2019), and DE-induced potentiation of mEPSCs is dependent on GluN2B-containing NMDARs (Bridi et al., 2018). Therefore, alterations in NMDAR function with prior visual experience may be responsible for the differential outcome of plastic changes induced by *ex vivo* stimulation. We found that blocking NMDARs containing the GluN2B subunit, which is known to be up-regulated in V1 following DE (Quinlan et al., 1999), or mGluR5s, which we recently reported to play a role in LE-induced metaplasticity (Chokshi et al., 2019), was rather ineffective at preventing *in vivo* spike train-driven decreases in mEPSC amplitudes. The molecular signaling of the homeostatic plasticity observed here will require further investigation. Nonetheless, our study demonstrates that stimulating the feedforward inputs to L2/3 with naturalistic activity patterns is sufficient to produce homeostatic synaptic depression even if the resulting plasticity is not identical to that produced with LE. Furthermore, our findings suggest that homeostatic synaptic plasticity, similar to Hebbian plasticity, is dependent on the metaplastic state of the circuit set by prior visual experience.

## Data Availability Statement

The original contributions presented in the study are included in the article, further inquiries can be directed to the corresponding author.

## Ethics Statement

The animal study was reviewed and approved by Johns Hopkins University Animal Care and Use Committee and University of Maryland Institutional Animal Care and Use Committee.

## Author Contributions

VC and BG performed *ex vivo* experiments and analyses. AD wrote the MATLAB code for spike pattern analysis. EN provided input on the spike pattern analysis program. AD and VC ran the spike pattern analysis program and statistics. CL and EQ performed *in vivo* single unit recordings and analyses. H-KL conceived the project. VC, BG, CL, EN, EQ, and H-KL wrote the manuscript. All authors contributed to the article and approved the submitted version.

## Conflict of Interest

The authors declare that the research was conducted in the absence of any commercial or financial relationships that could be construed as a potential conflict of interest.

## Footnotes

^1^https://github.com/heykyounglee/spike-train-analysis

## References

[B1] Abraham W. C. (2008). Metaplasticity: tuning synapses and networks for plasticity. Nat. Rev. Neurosci. 9:387. 10.1038/nrn2356 18401345

[B2] Bear M. F. Cooper L. N. Ebner F. F. (1987). A physiological basis for a theory of synapse modification. Science 237, 42–48. 10.1126/science.3037696 3037696

[B3] Beique J. C. Na Y. Kuhl D. Worley P. F. Huganir R. L. (2011). Arc-dependent synapse-specific homeostatic plasticity. Proc. Natl. Acad. Sci. U S A 108, 816–821. 10.1073/pnas.1017914108 21187403 PMC3021034

[B4] Bienenstock E. L. Cooper L. N. Munro P. W. (1982). Theory for the development of neuron selectivity: orientation specificity and binocular interaction in visual cortex. J. Neurosci. 2, 32–48. 10.1523/JNEUROSCI.02-01-00032.1982 7054394 PMC6564292

[B5] Bliss T. V. P. Gardner-Medwin A. R. (1973). Long-lasting potentiation of synaptic transmission in the dentate area of the unanaesthetized rabbit following stimulation of the perforant path. J. Physiol. 232, 357–574. 10.1113/jphysiol.1973.sp010274 4727085 PMC1350459

[B39] Bliss T. V. P. Lomo T. (1973). Long-lasting potentiation of synaptic transmission in the dentate area of the anaesthetized rabbit following stimulation of the perforant path. J. Physiol. 232, 331–356. 10.1113/jphysiol.1973.sp010273 4727084 PMC1350458

[B6] Bridi M. C. D. De Pasquale R. Lantz C. L. Gu Y. Borrell A. Choi S. Y.. (2018). Two distinct mechanisms for experience-dependent homeostasis. Nat. Neurosci. 21, 843–850. 10.1038/s41593-018-0150-0 29760525 PMC6019646

[B7] Chen L. Deng Y. Luo W. Wang Z. Zeng S. (2009). Detection of bursts in neuronal spike trains by the mean inter-spike interval method. Prog. Nat. Sci. 19, 229–235. 10.1016/j.pnsc.2008.05.027

[B8] Chokshi V. Gao M. Grier B. D. Owens A. Wang H. Worley P. F.. (2019). Input-specific metaplasticity in the visual cortex requires homer1a-mediated mglur5 signaling. Neuron 104, 736.e736–748.e736. 10.1016/j.neuron.2019.08.017 31563294 PMC6872932

[B9] Cooke S. F. Bear M. F. (2014). How the mechanisms of long-term synaptic potentiation and depression serve experience-dependent plasticity in primary visual cortex. Philos. Trans. R. Soc. Lond. B Biol. Sci. 369:20130284. 10.1098/rstb.2013.0284 24298166 PMC3843896

[B10] Cooper L. N. Bear M. F. (2012). The BCM theory of synapse modification at 30: interaction of theory with experiment. Nat. Rev. Neurosci. 13, 798–810. 10.1038/nrn3353 23080416

[B11] Desai N. S. Cudmore R. H. Nelson S. B. Turrigiano G. G. (2002). Critical periods for experience-dependent synaptic scaling in visual cortex. Nat. Neurosci. 5, 783–789. 10.1038/nn878 12080341

[B12] Dudek S. M. Bear M. F. (1992). Homosynaptic long-term depression in area CA1 of hippocampus and effects of N-methyl-D-aspartate receptor blockade. Proc. Natl. Acad. Sci. U S A 89, 4363–4367. 10.1073/pnas.89.10.4363 1350090 PMC49082

[B13] Espinosa J. S. Stryker M. P. (2012). Development and plasticity of the primary visual cortex. Neuron 75, 230–249. 10.1016/j.neuron.2012.06.009 22841309 PMC3612584

[B14] Fong M.-F. F. Newman J. P. Potter S. M. Wenner P. (2015). Upward synaptic scaling is dependent on neurotransmission rather than spiking. Nat. Commun. 6:6339. 10.1038/ncomms7339 25751516 PMC4355957

[B15] Gao M. Sossa K. Song L. Errington L. Cummings L. Hwang H.. (2010). A specific requirement of Arc/Arg3.1 for visual experience-induced homeostatic synaptic plasticity in mouse primary visual cortex. J. Neurosci. 30, 7168–7178. 10.1523/JNEUROSCI.1067-10.2010 20505084 PMC2881313

[B16] Goel A. Lee H. K. (2007). Persistence of experience-induced homeostatic synaptic plasticity through adulthood in superficial layers of mouse visual cortex. J. Neurosci. 27, 6692–6700. 10.1523/JNEUROSCI.5038-06.2007 17581956 PMC2601561

[B17] Goel A. Jiang B. Xu L. W. Song L. Kirkwood A. Lee H. K.. (2006). Cross-modal regulation of synaptic AMPA receptors in primary sensory cortices by visual experience. Nat. Neurosci. 9, 1001–1003. 10.1038/nn1725 16819524 PMC1905492

[B18] Goel A. Xu L. W. Snyder K. P. Song L. Goenaga-Vazquez Y. Megill A.. (2011). Phosphorylation of ampa receptors is required for sensory deprivation-induced homeostatic synaptic plasticity. PLoS One 6:e18264. 10.1371/journal.pone.0018264 21483826 PMC3069067

[B19] Guo Y. Huang S. De Pasquale R. Mcgehrin K. Lee H. K. Zhao K.. (2012). Dark exposure extends the integration window for spike-timing-dependent plasticity. J. Neurosci. 32, 15027–15035. 10.1523/JNEUROSCI.2545-12.2012 23100424 PMC3496177

[B20] Haider B. Schulz D. P. Hausser M. Carandini M. (2016). Millisecond coupling of local field potentials to synaptic currents in the awake visual cortex. Neuron 90, 35–42. 10.1016/j.neuron.2016.02.034 27021173 PMC4826437

[B100] Hebb D. O. (1949). The Organization of Behavior: Neuropsychological Theory. New York, NY: Wiley & Sons Inc.

[B21] Keck T. Keller G. B. Jacobsen R. I. Eysel U. T. Bonhoeffer T. Hubener M.. (2013). Synaptic scaling and homeostatic plasticity in the mouse visual cortex *in vivo*. Neuron 80, 327–334. 10.1016/j.neuron.2013.08.018 24139037

[B22] Kim J. Tsien R. W. (2008). Synapse-specific adaptations to inactivity in hippocampal circuits achieve homeostatic gain control while dampening network reverberation. Neuron 58, 925–937. 10.1016/j.neuron.2008.05.009 18579082 PMC2561251

[B23] Kirkwood A. Bear M. F. (1994). Hebbian synapses in visual cortex. J. Neurosci. 14, 1634–1645. 10.1523/JNEUROSCI.14-03-01634.1994 8126560 PMC6577523

[B24] Kirkwood A. Rioult M. C. Bear M. F. (1996). Experience-dependent modification of synaptic plasticity in visual cortex. Nature 381, 526–528. 10.1038/381526a0 8632826

[B25] Lee H. K. Kirkwood A. (2019). Mechanisms of homeostatic synaptic plasticity *in vivo*. Front. Cell. Neurosci. 13:520. 10.3389/fncel.2019.00520 31849610 PMC6901705

[B26] Lisman J. E. (1997). Bursts as a unit of neural information: making unreliable synapses reliable. Trends Neurosci. 20, 38–43. 10.1016/S0166-2236(96)10070-9 9004418

[B27] Murase S. Lantz C. L. Kim E. Gupta N. Higgins R. Stopfer M.. (2016). Matrix metalloproteinase-9 regulates neuronal circuit development and excitability. Mol. Neurobiol. 53, 3477–3493. 10.1007/s12035-015-9295-y 26093382 PMC4686372

[B28] Niell C. M. Stryker M. P. (2008). Highly selective receptive fields in mouse visual cortex. J. Neurosci. 28, 7520–7536. 10.1523/JNEUROSCI.0623-08.2008 18650330 PMC3040721

[B29] Perrett S. P. Dudek S. M. Eagleman D. Montague P. R. Friedlander M. J. (2001). LTD induction in adult visual cortex: role of stimulus timing and inhibition. J. Neurosci. 21, 2308–2319. 10.1523/JNEUROSCI.21-07-02308.2001 11264306 PMC6762413

[B30] Petrus E. Isaiah A. Jones A. P. Li D. Wang H. Lee H. K.. (2014). Crossmodal induction of thalamocortical potentiation leads to enhanced information processing in the auditory cortex. Neuron 81, 664–673. 10.1016/j.neuron.2013.11.023 24507197 PMC4023256

[B31] Petrus E. Rodriguez G. Patterson R. Connor B. Kanold P. O. Lee H. K.. (2015). Vision loss shifts the balance of feedforward and intracortical circuits in opposite directions in mouse primary auditory and visual cortices. J. Neurosci. 35, 8790–8801. 10.1523/JNEUROSCI.4975-14.2015 26063913 PMC4461685

[B32] Philpot B. D. Espinosa J. S. Bear M. F. (2003). Evidence for altered NMDA receptor function as a basis for metaplasticity in visual cortex. J. Neurosci. 23, 5583–5588. 10.1523/JNEUROSCI.23-13-05583.2003 12843259 PMC6741231

[B33] Philpot B. D. Sekhar A. K. Shouval H. Z. Bear M. F. (2001). Visual experience and deprivation bidirectionally modify the composition and function of NMDA receptors in visual cortex. Neuron 29, 157–169. 10.1016/s0896-6273(01)00187-8 11182088

[B34] Quinlan E. M. Olstein D. H. Bear M. F. (1999). Bidirectional, experience-dependent regulation of N-methyl-D-aspartate receptor subunit composition in the rat visual cortex during postnatal development. Proc. Natl. Acad. Sci. U S A 96, 12876–12880. 10.1073/pnas.96.22.12876 10536016 PMC23143

[B35] Rodriguez G. Mesik L. Gao M. Parkins S. Saha R. Lee H. K.. (2019). Disruption of NMDAR function prevents normal experience-dependent homeostatic synaptic plasticity in mouse primary visual cortex. J. Neurosci. 39, 7664–7673. 10.1523/JNEUROSCI.2117-18.2019 31413075 PMC6764196

[B36] Sun W. Dan Y. (2009). Layer-specific network oscillation and spatiotemporal receptive field in the visual cortex. Proc. Natl. Acad. Sci. U S A 106, 17986–17991. 10.1073/pnas.0903962106 19805197 PMC2764922

[B37] Turrigiano G. (2008). The self-tuning neuron: synaptic scaling of excitatory synapses. Cell 135, 422–435. 10.1016/j.cell.2008.10.008 18984155 PMC2834419

[B38] Turrigiano G. G. Leslie K. R. Desai N. S. Rutherford L. C. Nelson S. B. (1998). Activity-dependent scaling of quantal amplitude in neocortical neurons. Nature 391, 892–896. 10.1038/36103 9495341

